# Long-term Ashtanga yoga practice decreases medial temporal and brainstem glucose metabolism in relation to years of experience

**DOI:** 10.1186/s13550-020-00636-y

**Published:** 2020-05-14

**Authors:** June van Aalst, Jenny Ceccarini, Georg Schramm, Donatienne Van Weehaeghe, Ahmadreza Rezaei, Koen Demyttenaere, Stefan Sunaert, Koen Van Laere

**Affiliations:** 1 Nuclear Medicine and Molecular Imaging, Imaging and Pathology, UZ/KU Leuven, Herestraat 49, 3000 Leuven, Belgium; 2 Research Group Psychiatry, Neurosciences, University Psychiatric Center KU Leuven, Leuven, Belgium; 3 Adult Psychiatry, UZ Leuven, Leuven, Belgium; 4 Translational MRI, Imaging and Pathology, KU Leuven, Leuven, Belgium; 5 Radiology, UZ Leuven, Leuven, Belgium; 6 Nuclear Medicine, UZ Leuven, Leuven, Belgium

**Keywords:** Brain imaging, FDG, fMRI, PET/MR, Yoga, Glucose metabolism

## Abstract

**Background:**

Yoga is increasingly popular worldwide with several physical and mental benefits, but the underlying neurobiology remains unclear. Whereas many studies have focused on pure meditational aspects, the triad of yoga includes meditation, postures, and breathing. We conducted a cross-sectional study comparing experienced yoga practitioners to yoga-naive healthy subjects using a multiparametric 2 × 2 design with simultaneous positron emission tomography/magnetic resonance (PET/MR) imaging.

**Methods:**

^18^F-FDG PET, morphometric and diffusion tensor imaging, resting state fMRI, and MR spectroscopy were acquired in 10 experienced (4.8 ± 2.3 years of regular yoga experience) yoga practitioners and 15 matched controls in rest and after a single practice (yoga practice and physical exercise, respectively).

**Results:**

In rest, decreased regional glucose metabolism in the medial temporal cortex, striatum, and brainstem was observed in yoga practitioners compared to controls (*p* < 0.0001), with a significant inverse correlation of resting parahippocampal and brainstem metabolism with years of regular yoga practice (*ρ* < − 0.63, *p* < 0.05). A single yoga practice resulted in significant hypermetabolism in the cerebellum (*p* < 0.0001). None of the MR measures differed, both at rest and after intervention.

**Conclusions:**

Experienced yoga practitioners show regional long-term decreases in glucose metabolism related to years of practice. To elucidate a potential causality, a prospective longitudinal study in yoga-naive individuals is warranted.

## Background

Yoga combines the triad of meditation (*dhyana*), physical postures (*asana*), and focused breathing (*pranayama*). Originating in ancient India, the current 12-month prevalence of yoga practice is rising to about 9% in Western countries [1]. Apart from promotion of general health and well-being, yoga is advocated to ameliorate a variety of pathological conditions such as mood and anxiety disorders [2], where limited-to-moderate effects were found in symptom severity of depression and anxiety, comparable to medication and physical exercise interventions [3]. Nevertheless, based on a meta-analysis, no significant effects of yoga on symptoms of depression compared to treatment as usual were found [4].

In order to better assess potential benefits of yoga and predict to which patients it may be suited, a better understanding of the neurobiology of yoga is strongly needed. Only a limited number of neuroimaging studies have probed the effect of the yoga triad on the structural, functional, or molecular level using magnetic resonance imaging (MRI), positron emission tomography (PET), or single-photon emission tomography (SPECT). So far, most neuroimaging studies on yoga have focused on the meditational aspects rather than on the full triad. Overall, many studies suffer from variably defined control groups and a variety in yoga styles. The most consistently observed structural findings include increased gray matter density/volume in the hippocampus and insular cortex in experienced yoga practitioners compared to controls [5, 6], which may reflect changes in neurogenesis/synaptogenesis and changes in neuronal morphology [7]. Also, in experienced yoga meditation practitioners, increased functional connectivity was demonstrated, as measured with resting state functional MRI (rsfMRI), between the insula and frontal cortex [8] together with activity changes in the prefrontal cortex [9]. Based on DTI (diffusion tensor imaging), increased insular white matter integrity [10] was found. These changes have been linked to alterations in emotional/memory processing, strengthening of interoceptive and executive/control networks. On a molecular level, increased thalamic gamma-aminobutyric acid (GABA) levels were observed using magnetic resonance spectroscopy (MRS) in experienced yoga subjects immediately after a yoga practice [11]. In line with the latter, a leading hypothesis of the underlying neurobiological mechanism of yoga is that breathing exercises and baroreflex-promoting poses induce a shift in the parasympathetic nervous system and brain GABA levels through the vagal nerve [12, 13].

Based on this, we hypothesized that if yoga would regionally reduce neuronal activity through GABAergic inhibition, it should be measurable using [^18^F]fluorodeoxyglucose (FDG) PET, since glucose is considered the major source of energy in the brain and reflects predominantly glutamatergic neuronal-astrocyte activity [14]. To reduce heterogeneity in the current study, we focused on the effects of a widely practiced yoga style, *ashtanga*, that consists of a standard sequence of fixed poses, specific breathing, and meditative activity, implying low inter-subject variability in intervention performance, and compared that to a well-defined control group. Using simultaneous PET/MR imaging, we therefore conducted a 2 × 2 cross-sectional imaging study to assess FDG PET changes as primary objective. As secondary objective, alterations in structure, connectivity, and GABA-activity were explored using voxel-based morphometry (VBM), rsfMRI, DTI, and MRS.

## Materials and methods

### Participants

Twenty-five right-handed healthy volunteers between 24 and 52 years, participated in the study: a yoga group consisting of ten experienced yoga practitioners (age, 36.8 ± 7.0 years; 8 female/2 male) and a control group of 15 healthy controls (age, 34.6 ± 9.7 years; 13 female/2 male). Groups were matched for age, gender, education, and physical activity level. Experienced yoga practitioners had to practice ashtanga yoga since at least two years, three or more times a week. Otherwise, the same inclusion and exclusion criteria were used as detailed in Supplement 1 (Supplementary Materials and Methods).

The study was approved by the local university ethics committee and was conducted in full accordance with the latest version of the Declaration of Helsinki. All participants were recruited via response to advertisements on the local homepage of the hospital and social media. All participants provided written informed consent.

### Study design

**Fig. 1:**
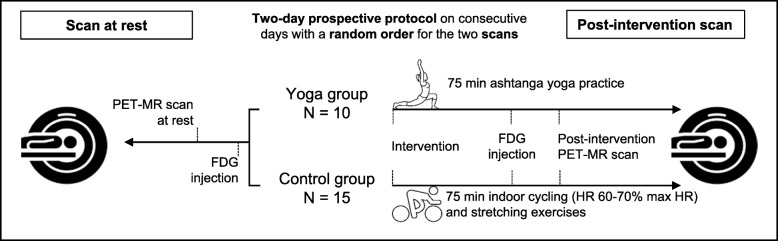
2 × 2 cross-sectional study design for both yoga and control groups, before and after intervention

In this cross-sectional study, a PET/MR scan at rest and post-intervention was performed on two consecutive days, with randomized order, as depicted in Fig. 1. All subjects fasted for three hours and were asked to abstain from yoga or stringent physical exercise twelve hours prior to FDG injection. The intervention for the yoga group included practicing the first series of *ashtanga* yoga sequences including a series of physical postures, breathing exercises, and meditation. The control group performed standardized physical exercises as their intervention.

### Image acquisition and reconstruction

An MRS scan was performed first (due to incompatibility of field-of-view placement) with 15 × 20 × 20 mm^3^ (6.0 cm^3^) and 15 × 15 × 15 mm^3^ (3.4 cm^3^) voxels positioned over left and right thalamus, and pregenual anterior cingulate cortex, respectively. GABA concentrations were derived by using a point-resolved spectroscopy sequence (PRESS sequence) (repetition time/echo time, 1500/35 ms, 192 averages).

For PET, subjects received an intravenous bolus injection of [^18^F]fluorodeoxyglucose (FDG) (mean ± SD, 117 ± 7 MBq) with a 20-min accumulation period in a quiet and dimly lit environment, either at rest or immediately after their intervention. The FDG emission scan was subsequently performed for 30 min (starting 53 ± 14 min postinjection, not significantly different between both groups and individual conditions).

Simultaneous to PET acquisition, the following MR sequences were acquired: 3D volumetric T1-weighted BRAVO sequence (plane, sagittal; TE, 3.2 ms; TR, 8.5 ms; TI, 450 ms; flip angle, 12°; receiver bandwidth, 31.25 kHz), zero-echo-time (ZTE) MR for attenuation correction (3D radial acquisition; flip angle, 0.8°; bandwidth, 62.5 kHz), diffusion tensor imaging (DTI) (TR, 12000 ms; TE, 85 ms; b0, 1500s/mm^3^; directions, 48; slices, 55), and resting-state functional MR (rsfMRI), using T2*-weighted gradient-echo planar imaging (GE-EPI) (TR, 1700 ms; TE, 23 ms; flip angle, 90°; number of volumes, 320). During the resting-state functional MR data acquisition, subjects were asked to close their eyes without falling asleep.

At the end of the PET acquisition, a single venous blood sample was collected to measure blood glucose concentration and the remaining FDG activity, to calculate absolute glucose consumption (Hunter method) [15].

PET data were rebinned in six frames of 5 min, and corrected for deadtime, randoms, scatter, and attenuation. An MR-based attenuation correction using the ZTE sequence was used [16]. PET images were reconstructed using OSEM (ordered subsets expectation maximization: 28 subsets, 4 iterations) algorithm, including time-of-flight information, resolution modeling, isotropic Gaussian post-smoothing with a FWHM (full width half maximum) of 4.5 mm, and corrected for time-of-flight offsets [17].

### PET processing

PET data were analyzed using SPM12 (Statistical Parametric Mapping, Wellcome Department of Imaging Neuroscience, London, UK) and for a volume-of-interest (VOI)-based analysis using PMOD software (v3.9, PMOD Inc, Zurich, Switzerland). PET data were corrected for motion and averaged to obtain a static FDG image. After realignment to the scan at rest and coregistration to T1-weighted MR image, spatial normalization (1 × 1 × 1 mm voxel size) to the Montreal Neurological Institute (MNI) space was done in SPM12. Before analysis, PET images were additionally smoothed using a Gaussian FWHM of 10 mm. Parametric images for regional cerebral metabolic rate of glucose (rCMRGlu) (mmol/l/min) were calculated based on a simplified kinetic model using blood glucose concentration and remaining FDG activity of a single venous sample (Hunter method). One yoga subject was excluded from this analysis due to a technical issue. For absolute or relative (normalization on whole brain counts), a relative gray matter analysis threshold of 80% of the mean was adopted to exclude extracerebral activity. Voxel-based findings were corroborated with a predefined VOI analysis using the N30R83 Hammers probabilistic atlas and AAL-merged atlas in PMOD [18, 19]. The AAL atlas allows for a more detailed delineation of the entire brainstem, encompassing VOIs for the midbrain, pons, and medulla, respectively.

Associations between FDG uptake and self-reported years of regular *ashtanga* yoga experience were also at the voxel and VOI level assessed.

### MR processing

For the voxel-based morphometry (VBM) analysis, the Computational Anatomy Toolbox (CAT12) [20], implemented in SPM12, was used. After segmentation, spatial normalization (DARTEL algorithm) and modulation, GM images were smoothed with a Gaussian kernel of 8 mm. Statistical unpaired *t* tests were conducted, with total intracranial volume (TIV) as confound, and an absolute threshold masking of 0.1 to avoid edge effects around borders between GM and WM.

Diffusion tensor images (DTI) were distortion corrected (FMRIB Software Library (FSL); University of Oxford, UK); Gibbs-rings, eddy-current, and motion artifacts were corrected using ExploreDTI [21]. Differences between the extracted fractional anisotropy (FA) and mean diffusivity (MD) values between both groups were assessed with a voxel-based analysis and atlas-based VOI analysis.

Resting state fMRI data were pre-processed and analyzed using the Conn toolbox v17 [22]. Due to the explorative nature of this study, multiple regions-of-interest (ROIs), including atlas regions and resting-state network nodes (default mode, salience, somatosensory, visual, dorsal attention, and language network) were included to perform a ROI-to-ROI seed-based analysis. rsfMRI data were explored at *p* < 0.05 (FDR analysis-level corrected). Additionally, an independent component analysis (ICA) was performed on the resting-state fMRI data to perform an ICA-to-voxel analysis to address differences between groups and conditions.

Proton-magnetic resonance spectroscopy data were processed and analyzed using JMRUI [23]. The QUEST algorithm was used for metabolite quantification with simulated short echo spectra (TE = 35 ms) of metabolites as prior knowledge, combined with a “Subtract” approach for background modeling.

### Statistical analysis

Data values are presented as mean ± SD and evaluated at the *p* < 0.05 level. Conventional statistical analyses were conducted in SPSS (v25, IBM, Corporation, Chicago, Illinois) or Prism (v5, GraphPad, San Diego, USA). Except for the voxel-based image analyses in the SPM/Conn toolbox, all numerical data sets were tested for normalized distributions and subsequent parametric or non-parametric analytical tests were chosen accordingly. Unless stated otherwise, voxel-based data were processed with the following thresholds: *P*_height_ = 0.001 (uncorrected), cluster extent of > 160 mm^3^, and FWE cluster corrected.

For the yoga subjects, the correlations between volume-of-interest-based glucose metabolism results and self-reported years of regular *ashtanga* practice were analyzed with a Spearman’s rank test.

## Results

### Demographics

In total, 10 experienced yoga subjects and 15 healthy controls were included in the study (Table 1). Groups did not significantly differ in age, gender, education, weekly physical activity level, or glycemia levels. The control group had a significantly higher BMI (24.8 ± 3.6) compared to the yoga group (21.2 ± 1.6).

**Table 1:** Subject demographics and activity levels

	Yoga subjects	Control subjects	*p* value
Gender (F/M)	8/2	13/2	0.66
Age (years)	36.8 ± 7.0	34.6 ± 9.7	0.55
Education (years)	15.6 ± 2.1	15.7 ± 1.4	0.85
Activity level (h/week)	5.5 ± 1.1	4.9 ± 3.2	0.58
Regular yoga practice (years)	4.8 ± 2.3	–	–
Glycemia (mmol/L)	4.9 ± 0.3	4.8 ± 0.4	0.93
BMI (kg/m^2^)	21.2 ± 1.6	24.8 ± 3.6	0.003^*^

Data is expressed as mean ± standard deviation, *BMI* body-mass index, *F* female, *M* male. ^*^indicates significant difference

### PET findings at rest

**Fig. 2:**
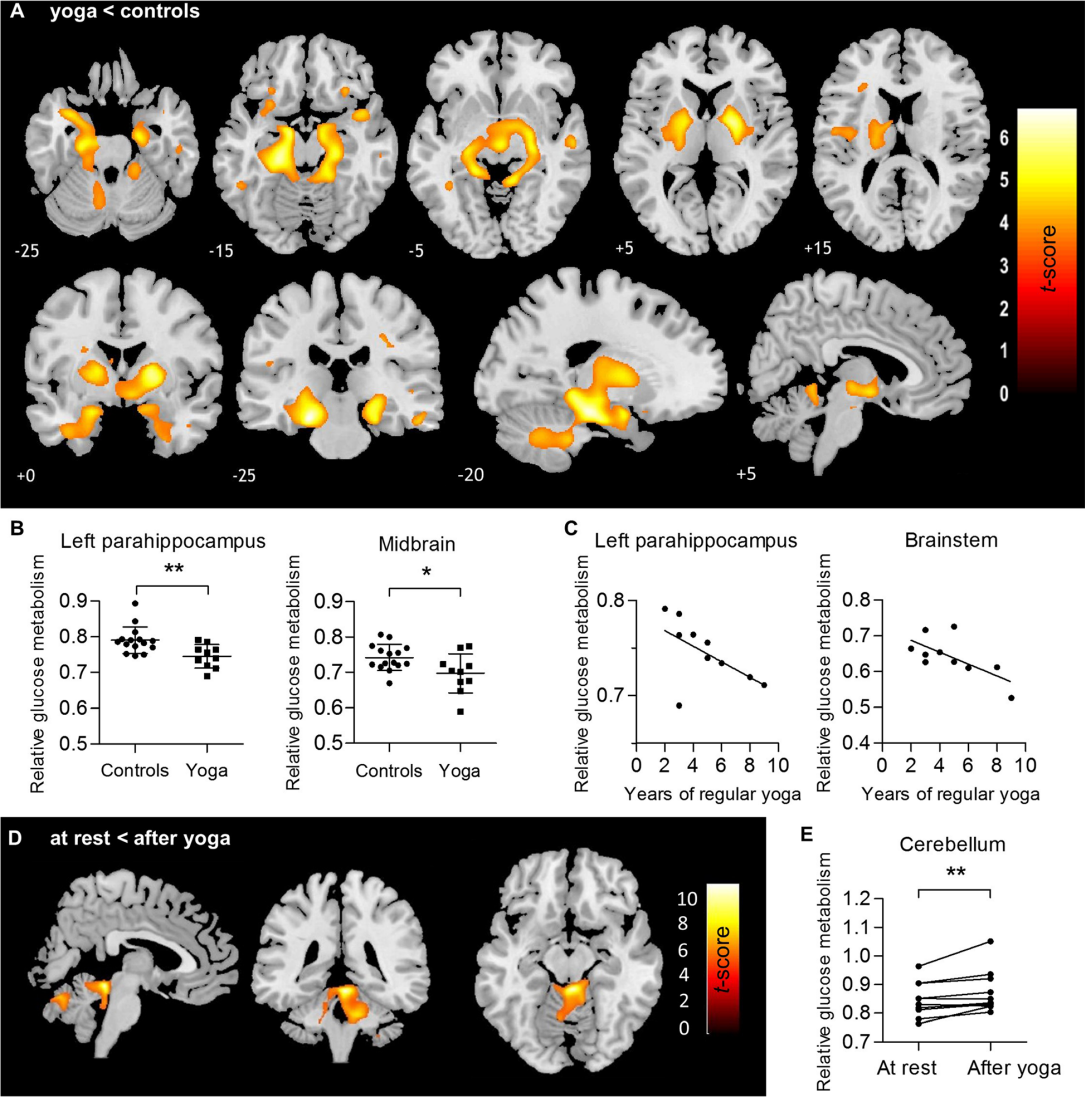
**a***t* statistical map (yoga < controls) of decreased relative glucose metabolism, overlaid on a T1-weighted MR template in neurological convention (*P*_*FW*E_ < 0.05 at cluster level, *P*_*height*_ < 0.001 uncorrected at voxel level, *K*_ext_ > 1.6 cm^3^). **b** Regional relative glucose metabolism in the left parahippocampus (*U* = 27.0, − 5.7%, *p* = 0.008) and midbrain (*t* = 2.4, − 6.0%, *p* = 0.024). **c** Negative correlation (*ρ* = Spearmann rank) between glucose metabolism and years of regular yoga practice, in left parahippocampus (*ρ* = − 0.63, *p* = 0.050) and in brainstem (*ρ* = − 0.64, *p* = 0.046). **d***t* statistical map (after yoga > at rest) of relative increased glucose metabolism, overlaid on a T1-weighted MR template in neurological convention. *P*_*FWE*_ < 0.05 at cluster level, *P*_*height*_ < 0.001 uncorrected at voxel level, and *K*_*ext*_ > 1.6cm^3^. **e** Regional relative glucose metabolism in the cerebellum (*W* = 49.0, 3.2%, *p* = 0.010). Significance is denoted as: *p < 0.05, **p < 0.01, uncorrected. *t t* test, *U* Mann-Whitney *U* test; *W* Wilcoxon signed rank test

No differences in absolute glucose metabolism (rCMRGlu) were found between the groups at rest. SPM group analysis of the relative FDG PET data showed a highly significant decrease in glucose metabolism at rest for the yoga practitioner group compared to controls in the hippocampus, parahippocampus, amygdala, insula, anterior midbrain, striatum (globus pallidus), and cerebellum (vermis, upper cerebellum), with a peak effect in the left parahippocampus of − 8.4% (*P*_*FWE*_ < 0.0001) (Fig. 2a and Supplementary Table S1). The VOI-based analysis confirmed this significant relative decreased glucose metabolism especially in the left parahippocampus (− 5.7%, *p* = 0.007) and in the midbrain (− 6.0%, *p* = 0.024) in the yoga group (Fig. 2b) (no Bonferroni correction applied). Averaged relative transverse FDG images for each group are given in Supplementary Figure S1.

Correlation analysis in the yoga group showed that decreased glucose metabolism in the parahippocampus (*ρ* = − 0.628, *p* = 0.05) and full brainstem VOI (*ρ* = − 0.640, *p* = 0.046) was inversely related to the years of regular *ashtanga* yoga practice (Fig. 2c).

### Post-intervention PET findings

No differences in absolute glucose metabolism (rCMRGlu) were found between the rest condition and after yoga. However, a regional relative increase in glucose metabolism in the cerebellum (peak effect 4.7%, *P*_*FWE*_ < 0.0001) was observed after a single yoga practice compared to the rest condition in the yoga group (Fig. 2d and Supplementary Table S2). This was confirmed in the VOI-based analysis for the whole cerebellum VOI (3.2%, *p* = 0.010) (Fig. 2e). In contrast, a single cycling/stretching intervention in controls did not result in detectable glucose metabolism changes. Averaged relative transverse FDG images for both groups after intervention are also supplied in Supplementary Figure S1.

### MR results

We did not observe significant differences in gray matter volume as measured with VBM. Both at rest and after intervention, no group differences or intervention interaction was found for the main rsfMRI networks (default mode, salience, somatosensory, visual, dorsal attention, and language network), nor for the ROI-to-ROI analyses. For MRS, GABA levels at baseline nor after intervention were different between groups (all *p* > 0.1). As for DTI, also no differences in regional WM FA and MD were found between the yoga and control group at baseline.

## Discussion

The primary objective of this study was to cross-sectionally determine differences in brain glucose metabolism between yoga practitioners and control subjects, at rest and after intervention. The main limbic regions where resting glucose metabolism was reduced in experienced yoga subjects included hippocampus, parahippocampus, and amygdala, encompassing key areas in mood and affect regulation [24, 25]. As for the amygdala, smaller right amygdala volumes have been found in yoga and meditation practitioners that were significantly correlated with years of practice [26]. Furthermore, in mindfulness meditation, the default mode network (DMN) is less active in experienced meditators [27]. The DMN encompasses the medial temporal subsystem including the hippocampus [27, 28]. Also, improved regulation of somatic and negative emotional arousal has been found, coupled to decreased glucose metabolism in the parahippocampus and insula [29, 30].

Decreased metabolism was also found in the left insula in the yoga group. The insula plays a pivotal role in interoception, body awareness, and viscero-emotional processing. It is involved during meditation, but also upon postural changes and slow paced breathing [12], its activity is associated to increased pain tolerance in yoga practitioners [31] and it has been linked to stronger functional connectivity with the frontal cortex upon meditation [8].

Symmetrical clusters of reduced metabolism were also observed in the upper cerebellum and vermis, as well as in the striatum. These motor function areas are involved in controlled movement, balance, and proprioception that are interrogated during various *asthanga* postures. On the other hand, controlled slow rate paced breathing has also been related to cerebellar and striatal changes related to cardiorespiratory control [12].

Changes in the brainstem in our study were predominantly located in the anterior to central midbrain area. The spatial resolution of the applied state-of-the-art time-of-flight PET with resolution recovery, is about 4 mm [32], which does not allow further detailed subregion differentiation of the altered metabolism in this complex nuclei-rich basal brain area. In the anterior/central midbrain, dopaminergic projection neurons from the ventral tegmental area are located. Increased dopamine tone with decreased desire for action has been observed during yoga nidra meditation [33]. Also, the median raphe with glutamatergic efferents is located in this cluster. Further high-resolution or specific neurotransmitter-based PET or detailed MR spectroscopy may further pinpoint the causative origin of this area.

Overall, the interpretation of lower baseline metabolism may be threefold. First, in line with the GABAergic stimulation hypothesis of yoga [11, 12], GABA-mediated cortical inhibition, and the breathing and baroreceptor response of yoga practice, may lead to increased cortical and subcortical inhibition, resulting in lower baseline neuronal activity [34]. Although FDG uptake is mainly driven by glutamatergic synapses and GABAergic and other neurotransmitters present a minor contribution, a slight decrease in FDG uptake due to elevated GABAergic cannot be ruled out. Secondly, in line with recent studies on brain energetics and network communication efficiency [35, 36], chronically a regionally more efficient glucose metabolism may be present. Thirdly, a pre-existing trait with reduced limbic resting activity may have been present in experienced yoga subjects. Stress reduction and desire for improved emotional well-being are the top outcomes of yoga in the vast majority of practitioners [1], and pre-existing stress or anxiety traits/disorders may in part be responsible for the current findings. Although the reason for starting yoga was not explicitly investigated in our population, psychometric variables were not different (data not shown). Also, decreased neuronal activity in medial temporal areas are also seen in neurodegenerative cognitive disorders although with much higher intensity differences, the functional neuroanatomy of the hippocampus and parahippocampus is determined by multiple circuits encompassing emotional memory and a richness of neurotransmitter systems, so we do not assume any implications on the cognitive level of the current findings.

Additional short-term metabolic effects include a relative increase in cerebellar glucose metabolism immediately after a single yoga session, in contrast to the observed decrease at rest in yoga subjects. This might reflect a prolonged effect of physical postures, breathing, and meditational components on the cerebellum.

In controls, the acute cycling/stretching intervention did not alter global or regional metabolism. Most previous studies on low to moderate intensity steady-state cycling or running paradigms have not found differences in global cerebral glucose metabolism or blood flow after single or acute exercising, but when FDG uptake was measured during the exercises global decreases were found [37], related to exercise intensity, up to about − 30%. Other substrates such as lactate may maintain neuronal activity in the acute setting [38, 39] and timing is important as during constant physical exercise, an increase in energy consumption is seen that returns to the resting level as the exercise continues [40]. As for the MRI measures, in neither rest nor activation setting, differences were found and can therefore not corroborate previous studies in specific yoga forms or meditation [8, 30]. Aside from the small sample and associated chance of type II errors, this negative result may also partly be explained by the relatively low sensitivity of the applied 8-channel coil.

Although most subjects in the yoga group indicated that the yoga session was similar to their general practice, the experimental setting may have disrupted the typical relaxed environment where yoga is practiced. The difference in BMI (Table 1) did not influence the observed FDG PET findings (data not shown). Finally, because of the wide variation in yoga styles ranging from low to high intensity styles, these results therefore not necessarily allow extrapolation to other yoga styles with varying relative contributions of physical postures, meditation, or breathing exercises [5]. Thus, larger group sizes and/or other specific styles of yoga are thus to be studied similarly to disentangle these various aspects in further detail.

## Conclusions

Brain glucose metabolism in medial temporal, insular, striatal, and midbrain is reduced in experienced yoga practitioners and regionally correlated with years of regular yoga practice, suggesting an interaction between *ashtanga* yoga practice and brain metabolism. Furthermore, cerebellar metabolism is increased after a single yoga practice in the experienced yoga practitioners. In contrast, no demonstrable alterations in MR-based measures were found in this study.

## Supplementary information

**Additional file 1:.** Supplementary Material and Methods. Supplementary Results. Figure S1 Average normalized FDG uptake for the control and yoga group, at rest and post intervention. Supplementary TABLE 1. Statistical parametric mapping analysis for decreased relative glucose metabolism in the yoga group compared to controls, at rest. Contrast: yoga < controls. Supplementary TABLE 2. Statistical parametric mapping analysis for differences in glucose metabolism at rest and after a single yoga practice in the yoga group. Contrast: after yoga > at rest .

## Data Availability

Please contact author for data requests.
