# Supplementary material for: Long-term Ashtanga yoga practice decreases medial temporal and brainstem glucose metabolism in relation to years of experience
Source: EJNMMI Res. 2020 May 14;10:50. doi: 10.1186/s13550-020-00636-y (PMC7225240; doi:10.1186/s13550-020-00636-y)
Supplement: Supplementary file 1 — Additional file 1:. Supplementary Material and Methods. Supplementary Results. Figure S1 Average normalized FDG uptake for the control and yoga group, at rest and post intervention. Supplementary TABLE 1. Statistical parametric mapping analysis for decreased relative glucose metabolism in the yoga group compared to controls, at rest. Contrast: yoga < controls. Supplementary TABLE 2. Statistical parametric mapping analysis for differences in glucose metabolism at rest and after a single yoga practice in the yoga group. Contrast: after yoga > at rest . [file 13550_2020_636_MOESM1_ESM.docx]

# Supplement 1

# Long-term Ashtanga yoga practice decreases medial temporal and brainstem glucose metabolism in relation to years of experience

Short running title: FDG PET/MR in yoga practitioners

June van Aalst^a^, Jenny Ceccarini^a^, Georg Schramm^a^, Donatienne Van Weehaeghe^a^, Ahmadreza Rezaei^a^, Koen Demyttenaere^b,c^, Stefan Sunaert^d,e^, Koen Van Laere^a,f^

^a^Nuclear Medicine and Molecular Imaging, Imaging and Pathology, UZ/KU Leuven, Belgium

^b^Research Group Psychiatry, Neurosciences, University Psychiatric Center KU Leuven, Belgium

^c^Adult Psychiatry, UZ Leuven, Belgium

^d^Translational MRI, Imaging and Pathology, KU Leuven, Belgium

^e^Radiology, UZ Leuven, Belgium

^f^Nuclear Medicine, UZ Leuven, Belgium

Corresponding author:

June van Aalst, Herestraat 49, 3000 Leuven, Belgium,

[June.vanaalst@uzleuven.be](mailto:June.vanaalst@uzleuven.be)
Tel: +3216349103

## Supplementary Material and Methods

**Participants**

Inclusion criteria for the active healthy controls: no previous yoga, meditation or martial arts experience; physically moderately active for at least three hours a week for over one year. Exclusion criteria: history of any major internal disease, severe head trauma or severe mental disorder, current use of psychotropic drugs, sedatives or sleep medication, current use of any illicit drugs. Participants were in good health according to their medical history, physical neurological examination and screening using the SCL-90 Symptom Checklist.

## Supplementary Results

## **Supplementary FIGURE 1**


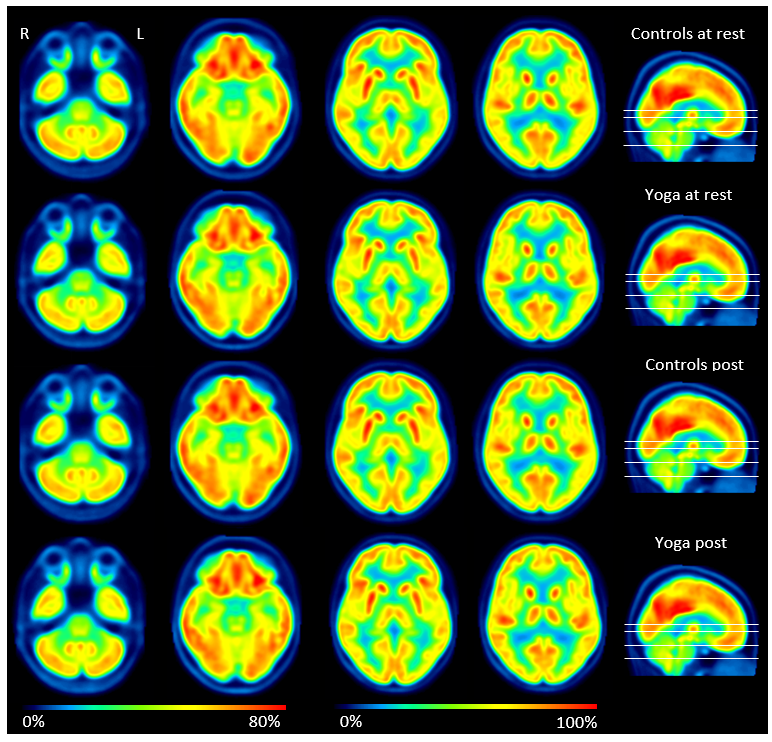


**Figure S1** Average normalized FDG uptake for the control and yoga group, at rest and post intervention.

## Supplementary Tables

**Supplementary TABLE 1.** Statistical parametric mapping analysis for decreased relative glucose metabolism in the yoga group compared to controls, at rest. Contrast: yoga < controls.

|  |  |  | Voxel |  | Peak voxel MNI | | | |  | Cluster peak intensity |  |  |
| --- | --- | --- | --- | --- | --- | --- | --- | --- | --- | --- | --- | --- |
| Cluster level | |  | level |  | coordinates | | | |  | Difference |  | Cluster location |
| *P*_FWE_ | K_EXT_ |  | *t* |  | x | y | z | |  | (%) |  |  |
| <0.0001 | 71178 |  | 6.5 |  | -22 | -24 | | -19 |  | -8.4 |  | L Parahippocampal gyrus |
|  |  |  | 5.8 |  | 24 | -7 | | 0 |  |  |  | R Pallidum |
|  |  |  | 5.8 |  | 20 | -1 | | 5 |  |  |  | R Pallidum |
|  |  |  | 5.7 |  | -13 | -7 | | -16 |  |  |  | L Hippocampal gyrus |
|  |  |  | 5.4 |  | 23 | -22 | | -20 |  |  |  | R Parahippocampal gyrus |
|  |  |  | 5.3 |  | 11 | -44 | | -8 |  |  |  | R Cerebellum 4 5 |
|  |  |  | 5.1 |  | 25 | -18 | | -7 |  |  |  | R Hippocampal gyrus |
|  |  |  | 5.1 |  | -30 | 7 | | -22 |  |  |  | L Temporal pole superior |
|  |  |  | 5.1 |  | 44 | 6 | | -12 |  |  |  | R Insular gyrus |
|  |  |  | 5.0 |  | 20 | -12 | | -23 |  |  |  | R Parahippocampal gyrus |

K_EXT_, cluster size extent (mm^3^); MNI, Montreal Neurological Institute; *P*_FWE_, familywise error corrected *P*-value.

**Supplementary TABLE 2.**  Statistical parametric mapping analysis for differences in glucose metabolism at rest and after a single yoga practice in the yoga group. Contrast: after yoga > at rest.

|  |  |  | Voxel |  | Peak voxel MNI | | | |  | Cluster peak intensity |  |  |
| --- | --- | --- | --- | --- | --- | --- | --- | --- | --- | --- | --- | --- |
| Cluster level | |  | level |  | coordinates | | | |  | Difference |  | Cluster location |
| *P*_FWE_ | K_EXT_ |  | *t* |  | x | y | z | |  | (%) |  |  |
| <0.0001 | 18643 |  | 11.7 |  | 3 | -38 | | -13 |  | +4.7 |  | R Vermis 3 cerebellum |
|  |  |  | 9.7 |  | -8 | -62 | | -17 |  |  |  | L Cerebellum 6 |
|  |  |  | 9.0 |  | -31 | -50 | | -44 |  |  |  | L Cerebellum 8 |
|  |  |  | 9.0 |  | -9 | -63 | | -38 |  |  |  | L Cerebellum 8 |
|  |  |  | 8.6 |  | -9 | -58 | | -25 |  |  |  | L Cerebellum 6 |
|  |  |  | 8.5 |  | 11 | -40 | | -32 |  |  |  |  |
|  |  |  | 7.9 |  | -26 | -60 | | -49 |  |  |  | L Cerebellum 8 |
|  |  |  | 7.2 |  | 4 | -74 | | -21 |  |  |  | R Vermis 7 |
|  |  |  | 7.1 |  | 14 | -33 | | -7 |  |  |  | R Lingual gyrus |
|  |  |  | 6.9 |  | -47 | -47 | | -38 |  |  |  | L Cerebellum Crus1 |

K_EXT_, cluster size extent (mm^3^); MNI, Montreal Neurological Institute; *P*_FWE_, familywise error corrected *P*-value.
